# Supplementary material for: Healthcare workers’ knowledge, attitude and practices during the COVID-19 pandemic response in a tertiary care hospital of Nepal
Source: PLoS One. 2020 Nov 6;15(11):e0242126. doi: 10.1371/journal.pone.0242126 (PMC7647104; doi:10.1371/journal.pone.0242126)
Supplement: Attachment — Submitted filename: PLOS-ONE REVISED PRIYANKA.docx [file pone.0242126.s002.docx]

**Healthcare workers’ knowledge, attitude and practices during the COVID-19 pandemic**

**response in a tertiary care hospital of Nepal**

Dil K Limbu1, Rano M Piryani 2, Avinash K Sunny3

*1Deparment of General Practice and Emergency Medicine, UCMS, Bhairahawa, Nepal*

*2Department of Internal Medicine, UCMS, Bhairahawa, Nepal*

*3Research Division, Golden Community, Lalitpur, Nepal*

**Corresponding author**

*Avinash K Sunny,*

*Research Division, Golden Community, Lalitpur, Nepal*

*Ph no: 977-9851122584, email: avinashkayastha@gmail.com*

**Abstract**

**Background**: To control the spread of ongoing COVID-19 infection, extremely important

measures need to be adopted. Healthcare worker’s adherence to prevention and control measures

is affected by their knowledge, attitudes, and practices (KAP) towards COVID-19. In this study,

we assessed the KAP among healthcare workers towards COVID-19 during the ongoing pandemic.

**Method:** A self-developed piloted KAP questionnaire was used among the recruited healthcare workers working for the COVID-19 response in the Universal College of Medical Sciences Teaching Hospital (UCMSTH), in Bhairahawa, Nepal. The knowledge questionnaire consisted of questions regarding the clinical characteristics, prevention and management of COVID-19. Assessment on attitudes and practices towards COVID-19 included questions on behavior and change in practices while working during this response. Knowledge scores were calculated and

compared by demographic characteristics and their attitude and practices towards COVID-19.

Data were analysed using bivariate statistics.

**Results:** A total of 103 healthcare workers participated in the study. The mean age of the

participants was 28.24 +/-6.11 years (range: 20-56); 60.2% were females; 61.2% were unmarried;

60.2% had medical degree and 39.8% were nursing staff. The mean knowledge score was

10.59±1.12 (range: 7-13) and it did not vary by demographic characteristics. Attitude was positive

for 53.4% participants with a mean knowledge score of 10.35±1.19 and negative for 46.6%

participants with a mean knowledge score of 10.88±0.98 (p=0.02). Practice was good (≥3 score)

for 81.5% participants with a mean knowledge score of 10.73±1.12 and practice was poor for

18.5% participants with a mean knowledge score of 10.46±1.13 (p=0.24). The attitude of the

participants improved with the increasing age of the participants (29.55±7.17, p=0.02).

**Conclusion**: There is comparably better knowledge regarding COVID-19 among healthcare

workers along with appropriate practices, however attitude was less optimistic with better

knowledge but more optimistic with higher age of the healthcare workers. Hence, protective

measures for healthcare workers in order to improve their attitude is necessary during the pandemic

response.

***Keywords***: attitude, COVID-19, healthcare worker, knowledge, Nepal, practice

**Introduction**

COVID-19 is a disease caused by the SARS-CoV-2 virus, first identified in the city of Wuhan, in

China's Hubei province in December 2019 (1). COVID-19 was previously known as 2019 novel

Corona virus (2019-nCoV) respiratory disease before the World Health Organization (WHO)

declared the official name as COVID-19 in February 2020 (2). In March 11, 2020, the World

Health Organization (WHO) declared the COVID-19 outbreak a pandemic (3). This ongoing

pandemic has been spreading very rapidly with more than 8.5 million confirmed infections and

more than 0.47 million deaths worldwide till June 22 2020 (GMT 01.18) (4).

Countries worldwide have used various control measures such as social distancing, hand washing,

closing of public transportation and public places, testing and tracing affected community (5). Like

many governments around the world, Nepal has also called for lockdown since 24rd March, 2020,

allowing only essential services to be available like hospitals, groceries and medical supplies and

frontline emergency services (6). In Nepal, the total number of confirmed infections stood at 9561

till June 22, 2020 (7).

Profound knowledge supports optimistic attitude and appropriate practice at work which in due

course helps deter the risk of infection (8). Healthcare worker's adherence to control measures is

affected by their knowledge, attitudes, and practices (KAP) towards COVID-19, hence it is

important to understand their knowledge and determine the factors that affect their attitudes and

practices in order to have adequate guidance and protection. Thus, this study aimed to assess the

KAP among healthcare workers towards COVID-19 during the ongoing pandemic.

**Method**

Study design and setting

A cross-sectional study was conducted from April 7, 2020 to May 7, 2020 at Universal College of

Medical Sciences Teaching Hospital (UCMSTH), Bhairahawa, Nepal which has recently stepped

its efforts to support government in its efforts towards COVID 19. UCMSTH is a teaching hospital

serving as a tertiary care center located in the southern west border of Nepal. All the healthcare

workers working in the hospital during the COVID-19 pandemic response were included in the

study.
***Data collection and management***

Data were collected from healthcare workers using a self-administered questionnaire to assess

KAP towards COVID-19. This questionnaire consisted of two parts with demographic information

and a self-developed piloted and pretested KAP questionnaire (2nd part) assessing knowledge

attitude and practice. Knowledge was measured through 13 questions: 5 regarding clinical

manifestation, 2 on the mode of transmission, and 6 for prevention and control of COVID-19 with

a true/false/don't know option. The reliability analysis of knowledge questionnaire with Cronbach's

alpha coefficient was found to be acceptable with an internal consistency of 0.76. Attitude and

practice was assessed through 5 questions each with a yes/no/don't know option. (Table 1)

Data were then entered into MS Excel and coded for anonymity. The entered data were exported

into Statistical Package for Social Sciences (SPSS) version 23 for analysis.

***Study variables***

***Demographic variables*** include age as continuous variable; gender as male and female; marital

status married and unmarried; education as certificate level with Auxiliary Nurse Midwife (ANM),

Proficiency Certificate Level (PCL) Nursing and Community Medical Assistant (CMA); Bachelor

in Nursing Science (BNS); graduate medical education with Bachelor of Medicine and Bachelor

of Surgery (MBBS); and post-graduate medical education with Doctor Medicine (MD), Master in

Dental Surgery (MDS) and Doctorate of Medicine (DM); and designation as Nursing staff,

Medical Intern, Medical Officer, Post Graduate (PG) Resident and Consultant.

***Knowledge scores*** were calculated by assigning 1 point to each correct answer and an

incorrect/unknown answer was assigned 0 points. The total knowledge score ranged from 0 to 13,

with a higher score signifying a better knowledge.

***Attitude*** was assessed as positive and negative. The average score on attitude questionnaire was

calculated and used as a cut off for positive and negative.

***Practice*** was assessed as good and poor. The average score on practice questionnaire was

calculated and used as a cut off for good and poor.

***Statistical analysis***

Descriptive statistics were calculated as frequency, percentage, mean and standard deviation (SD).

Data were analysed using Pearson chi-square test, Pearson correlation, independent t-test and oneway analysis of variance (ANOVA) test. At 95% Confidence Interval, p-value < 0.05 was

considered to be statistically significant.

***Ethical approval***

Ethical clearance was obtained from Institutional Review Committee (IRC) of Universal College

of Medical Sciences Teaching Hospital (UCMSTH). Informed written consent was taken from the

participant before inclusion in the study and confidentiality was maintained.

**Result**

The correct responses on KAP are mentioned in table 1. A total of 103 healthcare workers

participated in the study with a male to female ratio of 1:1.5. The mean age of the participants was

28.24±6.11 years (range: 20-56), 62 (60.2%) were females, 63 (61.2%) were unmarried, 62

(60.2%) had medical degree and 41 (39.8%) were nursing staff. The mean knowledge score was

10.59 +/- 1.12 (range: 7-13) with 81.5% correct answer rate. There was no significant correlation in the knowledge score of the participants and their mean age (p=0.13). The mean knowledge scores for male participants was 10.76±1.16 while for female participants 10.48±1.10, however the difference was not statistically significant (p=0.24). Married participants had mean knowledge

score of 10.78±1.00 while for unmarried participants it was 10.48±1.19, suggesting no statistically

significant difference (p=0.19). By education, the mean knowledge score was highest for MBBS

education with 10.85±1.28 (p=0.06) and by designation; it was highest for Resident with

11.00±0.86 (p=0.05), however the difference was not statistically significant. (Table 2)

The mean knowledge score varied significantly with the attitude of the participants (p=0.02) but it

did not vary with their practice (p=0.24). Attitude was positive (≥3 score) for 55(53.4%)

participants with a mean knowledge score of 10.35±1.19 and negative (<3 score) for 48(46.6%)

participants with a mean knowledge score of 10.88±0.98. Similarly, practice was good (≥3 score)

for 84(81.5%) participants with a mean knowledge score of 10.73±1.12 and practice was poor (<3

score) for 19(18.5%) participants with a mean knowledge score of 10.46±1.13. There was negative

correlation between knowledge and attitude (r=-0.313, p=0.001), however knowledge didn’t

correlate with practice (r=0.093, p=0.35). There was significant correlation between attitude and

practice (r=0.298, p=0.002). (Table 2 & Table 3)

**Table 1 Correct responses on KAP questionnaire (n=103)**

**Table 2 Knowledge scores of COVID-19 by demographic variables (n=103)**

**Table 3 Correlation between the Knowledge, attitude and practice scores (n=103)**

*****Pearson correlation

Association of attitude and practice of the participants towards COVID-19 with their

characteristics were assessed. There was no significant difference in the attitude of the participants

by their gender (p=0.66), marital status (p=0.51), education (p=0.42) or designation (p=0.28),

however the attitude varied significantly with the increasing age of the participants (p=0.02). The

practice of the participants was mostly good and did not differ by age (p=0.64), gender (p=0.82),

marital status (p=0.47), education (p=0.78) or designation (p=0.09). (Table 4)

**Table 4 Attitude and Practice towards COVID-19 by demographic variables (n=103)**

**Discussion**

This is a cross-sectional study conducted with objective to assess the KAP among healthcare

workers towards COVID-19 during the ongoing pandemic The findings of this study related to

overall correct answer (81.5%) are comparable with a similar study conducted in China which

reported that 89% healthcare workers surveyed demonstrated sufficient knowledge on COVID-19

(9). As this study was conducted during the national lockdown period in Nepal, healthcare workers

were quite aware of on most of the information related COVID-19 as part of being prepared for

the response to the ongoing pandemic. Despite the differences in demographic characteristics of

healthcare workers, the knowledge was better among all of the them. This is in contrast with the

studies suggesting differences in knowledge by the type of healthcare workers (10).

Overall, 53.4% of the healthcare workers had positive attitude towards COVID-19. This finding

is lower compared to that in other studies conducted in China. This speculation could probably

due to as only a half of them (50.5%) believed that Nepal could win the battle against COVID-19

and despite having family support (56.3%), they were less confident (45.6%) while at work.

because of anxiety and fear (78.6%) working with suspected patients and not all the doctors

(36.9%) from departments were actively involved in COVID-19 pandemic response. The attitude

of the healthcare workers was found to be similar across their different demographic

characteristics. This suggests that the notion was same for all in this pandemic situation, defying

the reports from other studies of varying attitude by the healthcare workers demographics (11).

Better knowledge of COVID-19 among the Healthcare workers did not correlate with their attitude

towards the disease while at work in this study. This finding is in contrast with other studies

reporting that knowledge directly affected their attitude and increased their confidence (9,10).

Knowledge is a prerequisite not only for establishing prevention beliefs and promoting positive

behaviours but also for forming positive attitudes towards disease for better effectiveness of their

coping strategies and behaviours to a certain extent (8). However, studies have reported that

protective measures not being in place could increase the chances of infection while well protected

emergency and other departments in the hospital had lower chances of infection (12). Healthcare

workers with higher age elicited positive attitude in this study. Similar was the case among

healthcare workers in other studies. Higher the age, longer is the experience in dealing with

emergencies ultimately demonstrating confidence and optimism. Hence, increasing age could be

the reason for positive attitude among the healthcare workers (13).

In this study, 81.5% of the healthcare workers practices was found to be appropriate and it did not

differ by their demographic characteristics or knowledge scores. However, the practice

significantly correlated with their attitude. Thus, poor practices can be linked to poor attitude and

it resulted so because very few (17.5%) were trained to work for COVID-19 patient, despite many

following practices such as social distancing (75.5%), wearing mask and gloves during hospital

practice (96.1%), infection protection measures (84.5%) and attending patient suspected with

COVID-19 (68.9%). This finding is comparable to a similar study in China which found 89.7% of

the healthcare workers followed correct practices regarding COVID-19 (9). Moreover, the

healthcare workers cannot neglect their own protection at work as they are the most vulnerable to

infection (14).

Limitations

This study has some limitations. The findings of this study should be cautiously used for

generalization since it depicts one hospital of Nepal. Additionally, KAP assessment may be

underestimated, hence further study is warranted.

Conclusion

This study found out that there is better knowledge regarding COVID-19 among healthcare

workers along with appropriate practices, however, attitude was less optimistic with better

knowledge and more optimistic with higher age of the healthcare workers. Healthcare workers

practice is directly correlated with their attitude. Hence, despite better knowledge, there is need

for more positive attitude and better practices to be in place. Thus, education and training on

protection and protective measures are required for subsequently improving positive attitude and

better practices at work during the COVID-19 pandemic response.
