# Supplementary material for: Healthcare workers’ knowledge, attitude and practices during the COVID-19 pandemic response in a tertiary care hospital of Nepal
Source: PLoS One. 2020 Nov 6;15(11):e0242126. doi: 10.1371/journal.pone.0242126 (PMC7647104; doi:10.1371/journal.pone.0242126)
Supplement: Attachment — Submitted filename: Edits on Paper Requested.docx [file pone.0242126.s003.docx]

Background: To control the spread of ongoing COVID-19 infection, extremely important measures need to be adopted (Elaborate or mention these measures and how they link with prevention and control measure adherence). Healthcare worker’s adherence to prevention and control measures is affected by their knowledge, attitudes, and practices (KAP) towards COVID-19. In this study, we assessed the KAP among healthcare workers towards the COVID-19 pandemic.

Method: A self-developed KAP questionnaire was administered to the recruited healthcare workers involved in the COVID-19 response at the Universal College of Medical Sciences Teaching Hospital (UCMSTH), in Bhairahawa, Nepal. The knowledge questionnaire consisted of questions regarding the clinical characteristics, prevention and management of COVID-19. Assessment on attitudes and practices towards COVID-19 included questions on behaviour and change in practices made towards COVID-19 response. Knowledge scores were calculated and compared by demographic characteristics and their attitude and practices towards COVID-19. Data were analysed using bivariate statistics.

Results: A total of 103 healthcare workers participated in the study. The mean age of the participants was 28.246.11 years (range: 20-56); 60.2% were females; 61.2% were unmarried; 60.2% had a medical degree and 39.8% were nursing staff. The mean knowledge score was 10.591.12 (range: 7-13) and it did not vary significantly when adjusted for demographic characteristics. Attitude was positive for 53.4% of the participants with a mean knowledge score of 10.35±1.19 and negative for 46.6% participants with a mean knowledge score of 10.88±0.98 (p=0.02). Practice was good (≥3 score) for 81.5% participants with a mean knowledge score of 10.73±1.12 and practice was poor for 18.5% participants with a mean knowledge score of 10.46±1.13 (p=0.24). The attitude of the participants improved with increasing age (29.55±7.17, p=0.02).

Conclusion: There is comparably better knowledge regarding COVID-19 among healthcare workers along with appropriate practices (This sentence does not make sense, needs to be put together better), however attitude was less optimistic with better knowledge but more optimistic with higher age of the healthcare workers. Hence, protective measures for healthcare workers in order to improve their attitude is necessary during the pandemic response. - please describe better, less optimism towards what? - catching COVID, death, disability??

Keywords: attitude, COVID-19, healthcare worker, knowledge, Nepal, practice

Introduction COVID-19 is a disease caused by the SARS-CoV-2 virus, first identified in the city of Wuhan, in China's Hubei province in December 2019 (1). COVID-19 was previously known as 2019 novel Corona virus (2019-nCoV) respiratory disease before the World Health Organization (WHO) declared the official name as COVID-19 in February 2020 (2). In March 11, 2020, the World Health Organization (WHO) declared the COVID-19 outbreak a pandemic (3). This ongoing pandemic has been spreading rapidly with more than 8.5 million confirmed infections and more than 0.47 million deaths worldwide as of June 22 2020 (GMT 01.18) (4).

Countries worldwide have used various control measures such as social distancing, hand washing, shutting public transportation and public places,and finally testing and tracing affected communities (5). Like many governments around the world, Nepal has also called for lockdown since 24rd March, 2020, allowing only essential services to be available like hospitals, groceries and medical supplies and frontline emergency services (6). In Nepal, the total number of confirmed infections stood at 9561 as of June 22, 2020 (7).

Profound knowledge supports optimistic attitude and appropriate practices at work which in due course helps deter the risk of infection (8). Healthcare worker's adherence to control measures is affected by their knowledge, attitudes, and practices (KAP) towards COVID-19. Therefore it is important to understand the knowledge of the medical providers and determine the factors that affect their attitudes and practices in order to have adequate practices and protection. Thus, this study aimed to assess the KAP among healthcare workers towards COVID-19 infections during the ongoing pandemic.

Method

Study design and setting

A cross-sectional study was conducted from April 7, 2020 to May 7, 2020 at Universal College of Medical Sciences Teaching Hospital (UCMSTH), Bhairahawa, Nepal. The hospital has recently stepped up its efforts to support the government in its fight towards COVID 19. UCMSTH is a teaching hospital serving as a tertiary care centre located in the southern west border of Nepal. All the healthcare providers working in the hospital during the COVID-19 pandemic response were included in the study.

Data collection and management

Data were collected from healthcare workers using a self-administered questionnaire to assess KAP towards COVID-19. This questionnaire consisted of two parts, the demographic information and a self-developed piloted and pretested KAP questionnaire. The KAP questionnaire assessed knowledge, attitude and practices of COVID-19 response.. Knowledge was measured using 13 true or false questions;. These questions assessed the provider’s knowledge on clinical manifestations, mode of transmission, prevention and control of COVID-19. The reliability analysis of the knowledge questionnaire with Cronbach's alpha coefficient was found to be acceptable with an internal consistency of 0.76. Attitude and practice were assessed using 5 questions with a yes/no/don't know option. (Table 1)

Collected Data was then entered into an MS Excel sheet and coded for anonymity. The entered data was exported into Statistical Package for Social Sciences (SPSS) version 23 for analysis.

Study variables

Demographic variables included age as a continuous variable; gender as male and female; marital status as married and unmarried; education as certificate level with Auxiliary Nurse Midwife (ANM), Proficiency Certificate Level (PCL) Nursing and Community Medical Assistant (CMA); Bachelor in Nursing Science (BNS); graduate medical education with Bachelor of Medicine and Bachelor of Surgery (MBBS); and post-graduate medical education with Doctor Medicine (MD), Master in Dental Surgery (MDS) and Doctorate of Medicine (DM); and designation as Nursing staff, Medical Intern, Medical Officer, Post Graduate (PG) Resident and Consultant. (too many running sentences, please use periods and shorter sentences)

Knowledge scores were calculated by assigning 1 point to each correct answer and a 0 to an incorrect/unknown answer. The total knowledge score ranged from 0 to 13, with a higher scores signifying better knowledge.

Attitude was assessed as positive and negative. The average score on the attitude questionnaire was calculated (what was the score) and used as a cut off for positive and negative. (Was anything above the score positive? - mention that here).

Practice was assessed as good and poor. The average score on the practice questionnaire was calculated and used as a cut off for good and poor. (Again please mention what the average score was, and the score above or below was labelled good or poor).

Statistical analysis

Descriptive statistics were calculated as frequency, percentage, mean and standard deviation (SD). Data were analysed using Pearson chi-square test, Pearson correlation, independent t-test and one way analysis of variance (ANOVA) test. At 95% Confidence Interval, p-value < 0.05 was considered to be statistically significant.

Ethical approval

Ethical clearance was obtained from the Institutional Review Committee (IRC) of Universal College of Medical Sciences Teaching Hospital (UCMSTH). Informed written consents were taken from the participants before inclusion in the study and confidentiality was maintained throughout.

Result

The correct responses on KAP are mentioned in table 1. A total of 103 healthcare workers participated in the study with a male to female ratio of 1:1.5. The mean age of the participants was 28.246.11 years (range: 20-56). 62 (60.2%) were females, 63 (61.2%) were unmarried, 62 (60.2%) had a medical degree and 41 (39.8%) were nursing staff. The mean knowledge score was 10.591.12 (range: 7-13) with 81.5% correct answer rate (what does this mean? Did 81.5% people answer correctly to 10 questions?). There was no significant correlation in the knowledge score of the participants and their mean age (p=0.13). The mean knowledge score for male participants was 10.76±1.16 while for female participants was 10.48±1.10,with no statistical significant (p=0.24). Married participants had a mean knowledge score of 10.78±1.00 while for unmarried participants it was 10.48±1.19, suggesting no statistically significant difference (p=0.19). By education, the mean knowledge score was highest for MBBS education with 10.85±1.28 (p=0.06) and by designation; it was highest for Resident with 11.00±0.86 (p=0.05) with no statistical significance

The mean knowledge score varied significantly with the attitude of the participants (p=0.02) but it did not vary with their practice (p=0.24). Attitude was positive (≥3 score) for 55(53.4%) participants with a mean knowledge score of 10.35±1.19 and negative (<3 score) for 48(46.6%) participants with a mean knowledge score of 10.88±0.98. Similarly, practice was good (≥3 score) for 84(81.5%) participants with a mean knowledge score of 10.73±1.12 and practice was poor (<3 score) for 19(18.5%) participants with a mean knowledge score of 10.46±1.13. There was negative correlation between knowledge and attitude (r=-0.313, p=0.001), however knowledge didn’t correlate with practice (r=0.093, p=0.35). There was significant correlation between attitude and practice (r=0.298, p=0.002). (Table 2 & Table 3)

Association of attitude and practice of the participants towards COVID-19 with their characteristics were assessed. There was no significant difference in the attitude of the participants by their gender (p=0.66), marital status (p=0.51), education (p=0.42) or designation (p=0.28), however the attitude varied significantly with the increasing age of the participants (p=0.02). The practice of the participants was mostly good and did not differ by age (p=0.64), gender (p=0.82), marital status (p=0.47), education (p=0.78) or designation (p=0.09). (Table 4)

Discussion

This is a cross-sectional study conducted with an objective to assess the KAP among healthcare workers toward the ongoing COVID-19 pandemic The findings of this study with relation to 81.5% providers answering correctly on the knowledge questionnaire is comparable with a similar study conducted in China which reported that 89% healthcare workers surveyed demonstrated sufficient knowledge on COVID-19 (9). As this study was conducted during the national lockdown period in Nepal, healthcare workers were quite aware of most of the information related to COVID-19 as part of being prepared for the response to the ongoing pandemic. Despite the differences in demographic characteristics of healthcare workers, the knowledge seemed to be on par with all of them. This is in contrast with other studies suggesting differences in knowledge with differing types of healthcare workers (10).

Overall, 53.4% of the healthcare workers had a positive attitude towards COVID-19. This finding is lower compared to that in other studies conducted in China. This attitude could probably be due to only half of the medical providers (50.5%) belief that Nepal could win the fight against COVID-19, Even with having family support (56.3%), they were less confident (45.6%) at work because of increased anxiety and fear (78.6%).(This sentence did not make sense, too many running sentences)The attitude of the healthcare workers was found to be similar across different demographic characteristics. This suggests that the notion was the same for all in this pandemic situation, defying the reports from other studies of varying attitudes by the healthcare workers demographics (11).

Having Better knowledge of COVID-19 among the Healthcare workers did not correlate with their attitude towards the disease while at work. This finding is in contrast with other studies reporting that knowledge directly affected their attitude and increased their confidence (9,10). Knowledge is a prerequisite not only for promoting preventive measures and promoting positive attitudes towards the fight against the disease(8). However, studies have reported that protective measures not being in place could increase the chances of infection while well protected departments in the hospital had lower chances of infection (12). Healthcare workers with higher age elicited a positive attitude in this study. Similar was the case among healthcare workers in other studies. Higher the age, longer is the experience in dealing with emergencies ultimately demonstrating confidence and optimism. Hence, increasing age could be the reason for positive attitude among the healthcare workers (13).

In this study, 81.5% of the healthcare workers' practices were found to be appropriate and it did not differ by their demographic characteristics or knowledge scores. (You mentioned earlier that knowledge scores were 81.5% correct here you mention that 81.5% did not differ by knowledge scores, I am confused - please explain this better). However, the practice significantly correlated with their attitude. poor practices can be linked to poor attitude,very few (17.5%) were trained to work for COVID-19 patient despite many following practices such as social distancing (75.5%), wearing mask and gloves during hospital practice (96.1%), infection protection measures (84.5%) and attending patient suspected with COVID-19 (68.9%). This finding is comparable to a similar study in China which found 89.7% of the healthcare workers followed correct practices regarding COVID-19 (9). Moreover, the healthcare workers cannot neglect their own protection at work as they are the most vulnerable to infection (14) - how is this last sentence relevant to the study?

Limitations

This study has some limitations - what are these limitations? Please mention and explain them here.The findings of this study should be cautiously used for generalization since it depicts one hospital of Nepal. Additionally, KAP assessment may be underestimated, - why do you think the KAP assessment was underestimated? hence further study is warranted.

Conclusion

This study found out that there is a positive correlation between knowledge regarding COVID-19 among healthcare workers along with appropriate clinical practices. However their attitude was less optimistic even with better knowledge. Higher optimism was seen with higher age. . Healthcare workers practice is directly correlated with their attitude. Hence, despite better knowledge, there is a need for a more positive attitude at the place of practice. ALso education and training on protection and protective measures are required for subsequently improving positive attitude and better practices at work during the COVID-19 pandemic response.

---------------

Too many running sentences, that are not properly explained

Your message is simple, but presented very complex with difficult sentences.
