# Supplementary material for: Healthcare workers’ knowledge, attitude and practices during the COVID-19 pandemic response in a tertiary care hospital of Nepal
Source: PLoS One. 2020 Nov 6;15(11):e0242126. doi: 10.1371/journal.pone.0242126 (PMC7647104; doi:10.1371/journal.pone.0242126)
Supplement: Attachment — Submitted filename: Response to Reviewers.docx [file pone.0242126.s004.docx]

Reviewers' comments:

Reviewer #1: Please see attached Document for recommended changes. There were many Grammar and Syntax errors that I corrected. It is a well done study and very appropriate in today's times. I enjoyed reading it. Great job!

Response: Thank you so much for appreciation and corrections as well. I have revised the corrections accordingly.

Reviewer #2: Please review my edits. Too many grammatical errors. Data is presented in a very complex way and too many running sentences. Please view the document with enabling the edits so you can see the revisions requested. Read my questions in () sentences to elaborate.

Response: Thank you so much for the review and suggestions. I have constructed simpler sentences as suggested and elaborated at the mentioned places.

Abstract:

Backgorund: “COVID-19 is an ongoing pandemic, for which appropriate infection prevention and control measures need to be adopted. Healthcare workers’ adherence to prevention and control measures is affected by their knowledge, attitudes, and practices (KAP) towards COVID-19.”

Conclusion: “There is comparably better knowledge regarding COVID-19 among healthcare workers. Appropriate practice correlates with better knowledge and positive attitude towards COVID-19 infection is seen with increasing age. Hence, training on protection and protective measures for having a positive attitude among healthcare workers is necessary against the fight with COVID-19 infection.”

Method section:

Study variables

***“Attitude*** was assessed as positive and negative. The average score on the attitude questionnaire was calculated and used as a cut off for positive and negative. The average score was 3 and scores below 3 was labelled negative.”

***“Practice*** was assessed as good and poor. The average score on the practice questionnaire was calculated and used as a cut off for good and poor. The average score was 3 and scores below 3 was labelled poor.”

Limitations:

***“***The findings of this study should be cautiously used for generalization since it depicts one hospital in Nepal. Additionally, practices being self-reported may not be actual; hence further study is warranted.”
